# Body Image Dissatisfaction and Risk of Eating Disorders Among University of Sharjah Students

**DOI:** 10.7759/cureus.104113

**Published:** 2026-02-23

**Authors:** Rahaf M Yousif Ahmed, Mohammed Naji AlAbsi, Samaha Kanden Mohammed, May Hussein Hassan, Omar Ahmad Al Mohdi, Noor Abdulla Al Kindi, Sama Hany Al Refaei, Amna Khalid

**Affiliations:** 1 College of Medicine, Burjeel Royal Hospital, Al Ain, ARE; 2 College of Medicine, Sheikh Khalifa Medical City, Abu Dhabi, ARE; 3 College of Medicine, Dubai Health, Dubai, ARE; 4 College of Medicine, Burjeel Medical City, Abu Dhabi, ARE; 5 College of Medicine, Khorfakkan Hospital, Sharjah, ARE; 6 College of Medicine, Al Qassimi Hospital, Sharjah, ARE; 7 College of Medicine, Mirdif Hospital, Dubai, ARE; 8 Family and Community Medicine and Behavioural Sciences, University of Sharjah, Sharjah, ARE

**Keywords:** body image dissatisfaction, covid-19 lockdown impact, eating attitudes, middle east/uae, psychosocial factors, social media influence, university students

## Abstract

Background and aim

Body image significantly influences an individual’s self-esteem and general mental well-being. A negative body image, defined as the discrepancy between one's real physique and their perceived ideal physique, can lead to considerable psychological and behavioral problems, such as body dissatisfaction and maladaptive eating habits. The primary purpose of this study was to evaluate the connection between body image dissatisfaction and eating attitudes among students at the University of Sharjah, with the aim of providing region-specific evidence to guide future health promotion programs targeting young people.

Methodology

In this cross-sectional study, data collection was carried out between January 2022 and March 2022, which was conducted through an online questionnaire, a text version of which is available in Appendix. Participants were recruited from multiple colleges within the University of Sharjah. In all, a total of 311 participants fulfilled the inclusion criteria. This study incorporated body dissatisfaction, body appreciation, and eating disorder (ED) risk, which were assessed using standardized questionnaires. Lastly, data were analyzed using SPSS Statistics version 23 (IBM Corp., Armonk, New York, USA).

Results

The results of the study revealed that out of the 311 individuals, the majority (65.6%, n = 204) had minimal to no risk of developing an eating disorder (ED) and reported high levels of body appreciation, indicating a low body dissatisfaction rate. There was no significant association between sex and body image dissatisfaction, with both males and females having similar ED risk (32.3% vs. 35.3%, p-value = 0.603). In contrast, some psychological factors were strongly associated with ED risk. Participants who reported that social media influenced their body perception had a higher incidence of ED (40.3%, n = 95; p-value = <0.001). Similarly, those who felt pressure to conform to social media beauty standards (50.0%, n = 79; p-value = <0.001) and those who received appearance-related remarks from peers or family members (41.2%, n = 87; p-value = <0.001) also demonstrated significantly higher ED risk. Lastly, participants who reported heightened awareness of their eating habits during the COVID-19 lockdown had a significantly higher risk of ED than those who reported no change (43.2%, n = 92 vs. 15.3%, n = 15; p-value = <0.001).

Conclusion

Despite high overall levels of body appreciation, a substantial proportion of the participants were at risk of eating disorders. Eating disorder risk and degree of body dissatisfaction were significantly associated with sociocultural factors, including social media influence, perceived pressure to conform to cultural beauty standards, pressure from family/peers due to appearance-related remarks, and the COVID-19 lockdown. The findings of our study underscore the need for preventive and educational initiatives that promote healthy body image and awareness of the potential psychological and behavioral consequences of unrealistic beauty standards.

## Introduction

Body image plays a pivotal role in shaping an individual’s self-esteem and overall mental health. A negative body image, defined as the disparity between one’s actual body and their perceived ideal, can result in significant psychological and behavioral challenges, including body dissatisfaction and maladaptive eating attitudes. Among university students, the pressure to conform to societal and cultural standards of beauty, often perpetuated by social media and external influences, can be understood through frameworks such as Social Comparison Theory [1]. This theory presumes that individuals evaluate themselves relative to perceived ideals. Additionally, the Tripartite Influence Model [2] highlights sociocultural influences as key drivers of body dissatisfaction and disordered eating. These factors may exacerbate the risk of body image dissatisfaction (BID) and contribute to disordered eating behaviors.

Body image dissatisfaction is a growing concern globally, with evidence indicating rising prevalence and strong associations with disordered eating patterns, low self-esteem, and other psychological comorbidities [3-6]. Although the phenomenon is well-documented in Western contexts, there is limited literature on its prevalence and implications within Middle Eastern student populations. Existing regional studies in the United Arab Emirates (UAE) and broader Middle East and North Africa (MENA) region have often focused on single-sex samples or specific subpopulations, and have reported mixed findings regarding the association between body image dissatisfaction and disordered eating [7-10]. Earlier work among Emirati female university students demonstrated a positive association between body image dissatisfaction and disordered eating attitudes [9], though findings were limited to female samples. This contrasts with more recent findings in a study of UAE female students [10], which did not observe a direct link between body dissatisfaction and disordered eating behaviors. Regional findings regarding gender differences in body image dissatisfaction also remain inconsistent; for instance, a study among UAE university students reported higher BID among males than females [11], contrasting with patterns frequently observed in Western literature. Together, these gaps highlight the need for comprehensive studies that are gender-inclusive, assess multiple contributing factors, and examine the relationship between body image dissatisfaction and eating attitudes in the UAE context.

Therefore, the primary objective of the present study was to explore the association between body image dissatisfaction and eating attitudes among students at the University of Sharjah (UOS). Secondary objectives included identifying the various sociocultural and environmental factors contributing to these issues, such as social media, culture, peer/family remarks, and the COVID-19 lockdown, which has been shown to have heterogeneous effects on body image and disordered eating outcomes [12]. It was hypothesized that higher levels of body image dissatisfaction would be associated with an increased risk of disordered eating and that sociocultural factors would be significantly related to both. Examining these relationships among a diverse UAE university student population, this study aims to provide region-specific evidence to guide future health promotion initiatives targeting young adults.

This article was previously presented as an abstract at the 2024 EHS International Mental Health Conference on October 4, 2024, and the 5th AHS Research and Education Conference on December 13, 2024.

## Materials and methods

Study design and setting

This study was conducted as a cross-sectional observational study among undergraduate students enrolled at the University of Sharjah, Sharjah, United Arab Emirates. Students from multiple colleges within the university were targeted. Data collection was carried out between January 2022 and March 2022.

Study population and sampling

The study population consisted of currently enrolled undergraduate students at the University of Sharjah. Participants were recruited using a non-probability volunteer sampling method through an electronically shared online questionnaire, a text version of which is available in Appendix.

Sample Size Calculation

The sample size was calculated using Cochran's formula [13], and an expected prevalence of 40% was adopted based on a similar previous study [11], with a 95% confidence level. The calculation was adjusted for the finite population size of the university, and a total of 311 participants met the inclusion criteria and were included in the final analysis.

Participant Distribution

Participants were recruited from multiple colleges within the university. The distribution across colleges was as follows: Medicine (57.6%), Health Sciences (10.3%), Engineering (7.7%), Dental Medicine (5.8%), Pharmacy (4.5%), Business Administration (3.5%), and other colleges (10.6%). This distribution demonstrates a marked predominance of medical students in the study sample.

In addition, female students constituted the majority of participants. This imbalance was identified early in the study and likely reflects higher participation rates among health-related disciplines and greater survey engagement among female students, particularly in studies addressing body image and eating-related behaviors. Consequently, this non-proportional representation may introduce sampling bias and limit the generalizability of the findings to the broader university student population. This limitation was considered during the interpretation of the results.

Eligibility criteria

Inclusion criteria included enrollment as an undergraduate student at the University of Sharjah at the time of the study and voluntary participation. Exclusion criteria comprised incomplete questionnaire responses and withdrawal prior to submission.

Data collection instrument

Data were collected using a self-administered online questionnaire consisting of 12 total questions, three of which were validated assessment instruments. The questionnaire consisted of five main sections: demographic characteristics, body image dissatisfaction [14], body appreciation score [15], Eating Attitudes Test-26 (EAT-26) [16], and psychosocial factors. A total of 311 participants fulfilled the inclusion criteria and were included in the final analysis.

Measures

Measures included validated assessment scales as well as researcher-developed items and calculated indices. Body dissatisfaction was assessed by calculating the discrepancy between participants' perceived actual and ideal body images, which they selected from provided images of the figure rating scale [14]. Body appreciation was assessed using the Body Appreciation Scale-2 (BAS-2) [15], a ten-item instrument rated on a five-point Likert scale. Eating disorder risk was evaluated using the Eating Attitudes Test-26 (EAT-26) [16].

For analytical purposes, eating disorder risk was analyzed as a categorical variable using the standard EAT-26 [16] cutoff score of ≥20 to classify participants as being at high risk for eating disorders. This approach was selected to enhance clinical relevance and ensure comparability with existing literature and original validation studies, in which the EAT-26 [16] is primarily employed as a screening tool rather than as a continuous outcome measure.

Questionnaire Administration

The questionnaire was administered in English and distributed electronically via a link to Google Forms. Responses were collected anonymously. A text version of the administered questionnaire can be found in Appendix.

Reliability and internal consistency

The internal consistency of the psychometric instruments used in the study was assessed using Cronbach's alpha. Both the Body Appreciation Scale (BAS-2) [15] and the Eating Attitudes Test (EAT-26) [16] demonstrated excellent internal consistency in the present sample (Cronbach's α = 0.93 for both scales). Other study variables were derived from single-item measures, calculated indices, or categorical questions and, therefore, were not subject to internal consistency analysis.

Variables

The primary variables of interest were body dissatisfaction score (BDS) and eating disorder risk. Secondary variables included body appreciation score, sex, perceived influence of social media on body image, influence of remarks from family or social circles, perceived cultural pressure to conform to beauty standards, perceived need to take extreme measures to achieve an ideal body type, and perceived changes in body image and eating behaviors during the COVID-19 pandemic.

Statistical analysis

Statistical analysis was performed using IBM SPSS Statistics version 23 (IBM Corp., Armonk, NY). Descriptive statistics were used to summarize participant characteristics and questionnaire responses. Normality of continuous variables was assessed using appropriate statistical tests; given the non-normal distribution and ordinal nature of questionnaire-derived scores, non-parametric tests were applied.

Associations between continuous variables were examined using Spearman's rank correlation coefficient. Associations between categorical variables were assessed using the chi-square test. Differences in body dissatisfaction scores between two independent groups were analyzed using the Mann-Whitney U test, while comparisons involving more than two independent groups were conducted using the Kruskal-Wallis test. Valid percentages have been calculated by eliminating incomplete responses from the denominator and representing each category as a fraction of the total number of valid responses. Cumulative percentages were derived by consecutively summing legitimate percentages across ranked response categories. A p-value of less than 0.05 was considered statistically significant.

Ethical considerations

Ethical approval was obtained from the Research Ethics Committee of the University of Sharjah (REC-22-02-04-S). The study was conducted in accordance with the approved research protocol. Participation was voluntary, and informed implied consent was obtained prior to questionnaire completion. No personally identifiable information was collected.

## Results

Participant characteristics

A total of 311 students from the University of Sharjah were enrolled in the study (Table 1). Most participants were female (70.0%, n = 218), with males making up 30.0% (n = 93) of the sample. Participants were between 17 and 24 years old, the majority of whom were 20 years old (37.9%, n = 118). Approximately 90% (n = 280) were of Arab nationality. More than half of the participants were enrolled in the College of Medicine (57.6%, n = 179), followed by the College of Health Sciences (10.3%, n = 32) and Engineering (7.7%, n = 24), with other colleges making up the remaining total. 

**Table 1:** Participant characteristics-sex, age, nationality, and college.

Variable	Frequency	Percent	Valid percent	Cumulative percent
Sex				
Male	93	29.9	29.9	29.9
Female	218	70.1	70.1	100.0
Total	311	100.0	100.0	-
Age (recoded)				
<20 years	109	35.0	35.0	35.0
20 years	118	37.9	37.9	73.0
>20 years	84	27.0	27.0	100.0
Total	311	100.0	100.0	-
Nationality groups				
UAE	58	18.6	18.8	18.8
Arabs	218	70.1	70.6	89.3
Non-Arabs	33	10.6	10.7	100.0
Total (valid)	309	99.4	100.0	-
Missing (system)	2	0.6	-	-
Total	311	100.0	-	-
College currently enrolled in				
Arts, humanities, and social sciences	4	1.3	1.3	1.3
Business administration	11	3.5	3.5	4.8
Engineering	24	7.7	7.7	12.5
Health sciences	32	10.3	10.3	22.8
Law	4	1.3	1.3	24.1
Fine arts and design	3	1.0	1.0	25.1
Communication	5	1.6	1.6	26.7
Medicine	179	57.6	57.6	84.2
Dental medicine	18	5.8	5.8	90.0
Pharmacy	14	4.5	4.5	94.5
Sciences	9	2.9	2.9	97.4
Computing and information	8	2.6	2.6	100.0
Total	311	100.0	100.0	-

Body dissatisfaction and body appreciation

**Figure 1:**
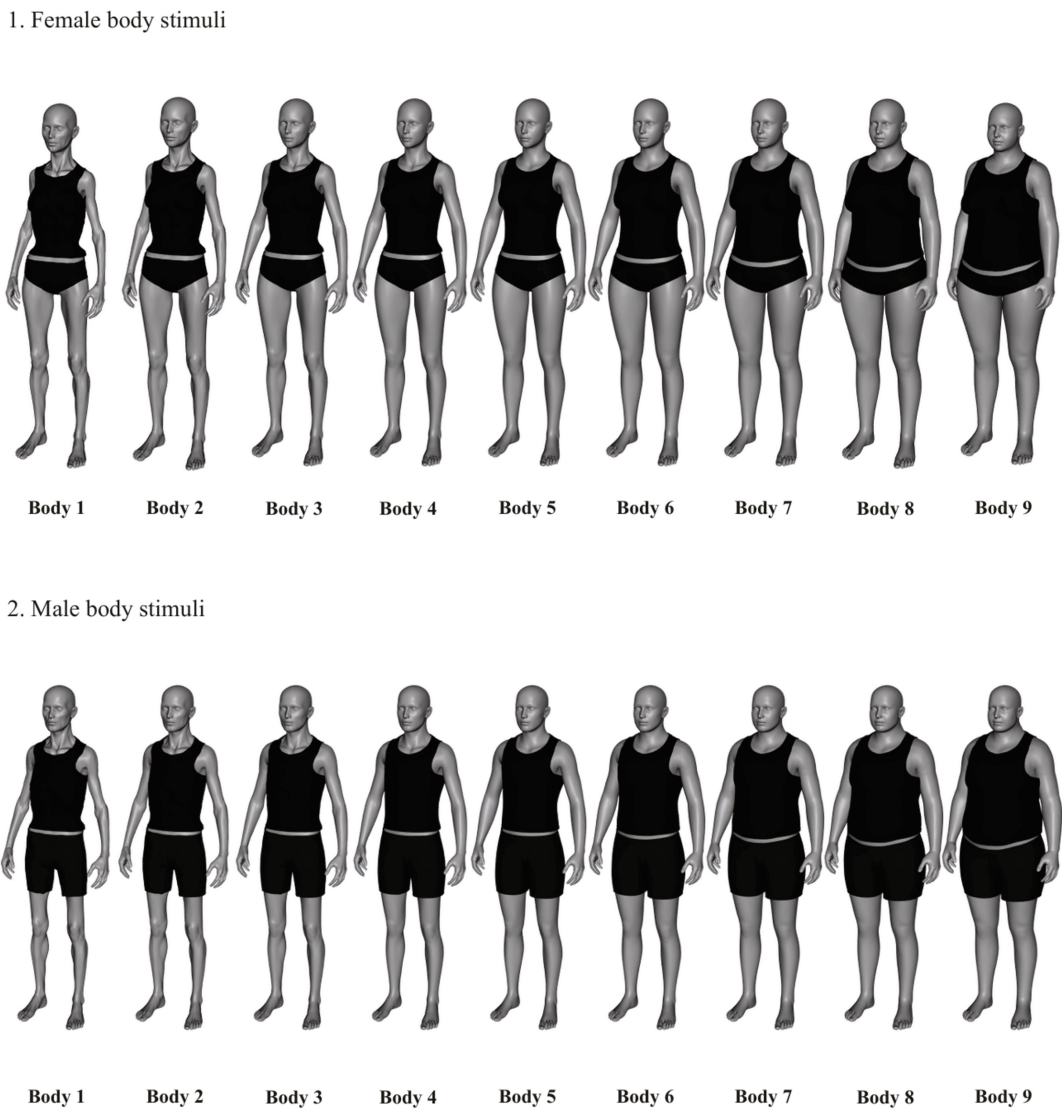
Figure rating scale of body image dissatisfaction (BID). Figure reproduced from Mutale et al. [14], licensed under a Creative Commons Attribution 4.0 International License (CC BY 4.0) (https://creativecommons.org/licenses/by/4.0/).

**Figure 2:**
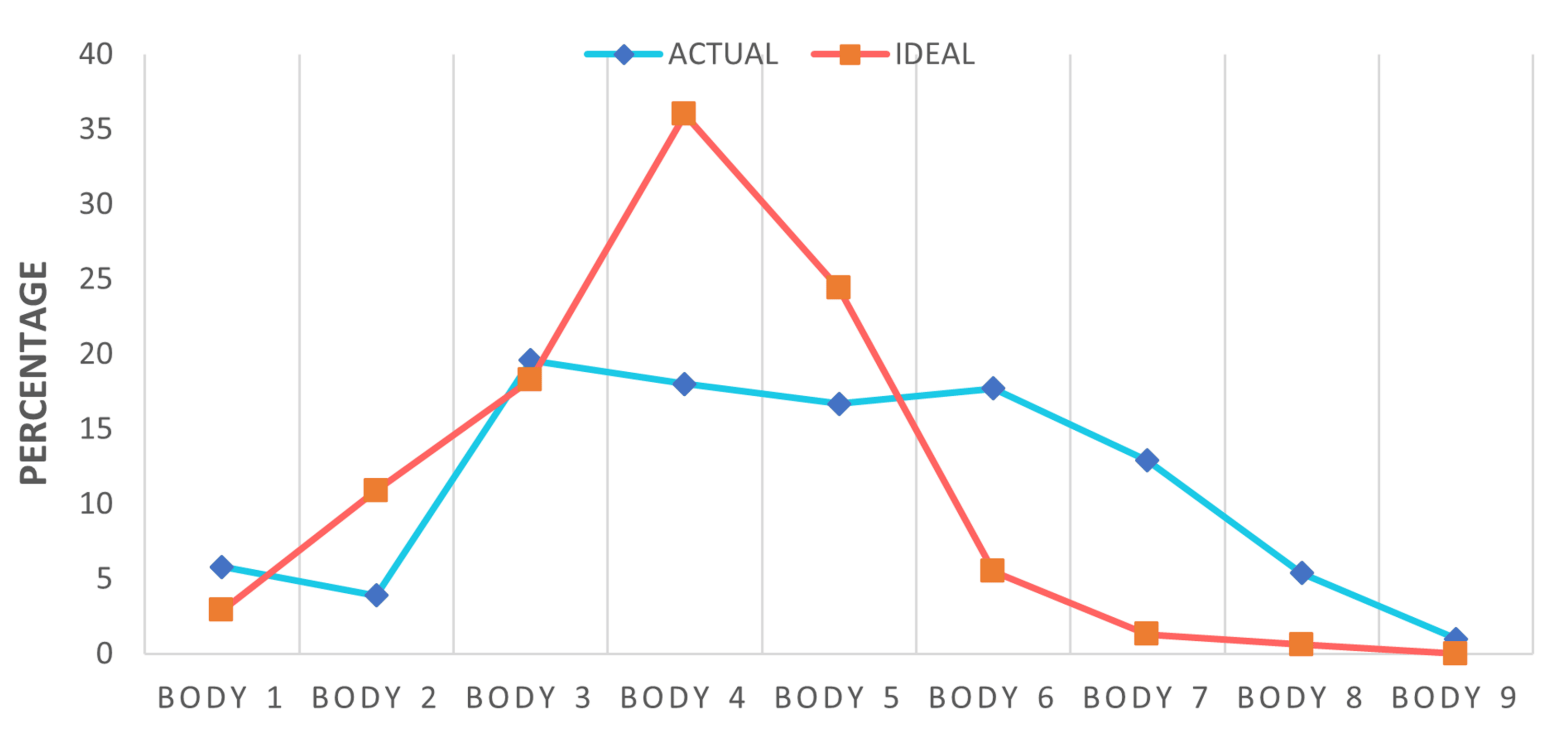
Perceived actual body image versus perceived ideal body image distribution. Body numbers refer to images on the figure rating scale [14].

**Figure 3:**
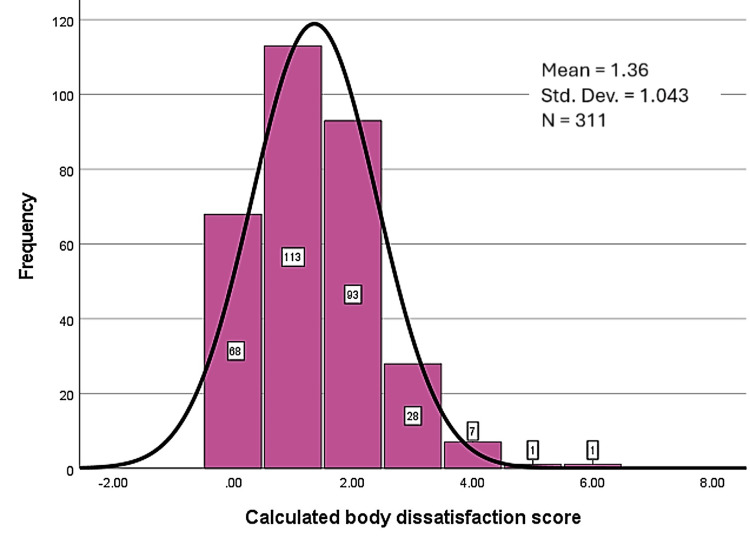
Histogram of body dissatisfaction scores. Body dissatisfaction scores were calculated based on the gap between perceived actual body image and perceived ideal body image (Figure 2).

Body dissatisfaction was assessed using sex-specific figure rating scales [14] (Figure 1), with a greater weight appearance in higher numbers. While 84.9% (n = 264) of participants chose between bodies 3-7 presented on the figure rating scales [14] (Figure 1) to represent their bodies, these numbers alone don't reveal dissatisfaction. The degree of dissatisfaction was measured separately using the gap between their perceived and ideal figures (Figure 2). The average body dissatisfaction score (BDS) was 1.36, indicating generally low dissatisfaction across our sample, and the majority of the participants scored between 0 and 3, as seen in the histogram below (Figure 3). Higher levels of dissatisfaction were uncommon, with only 2.3% (n = 7) scoring 4 and fewer than 1% (n = 2) scoring 5 or 6.

**Figure 4:**
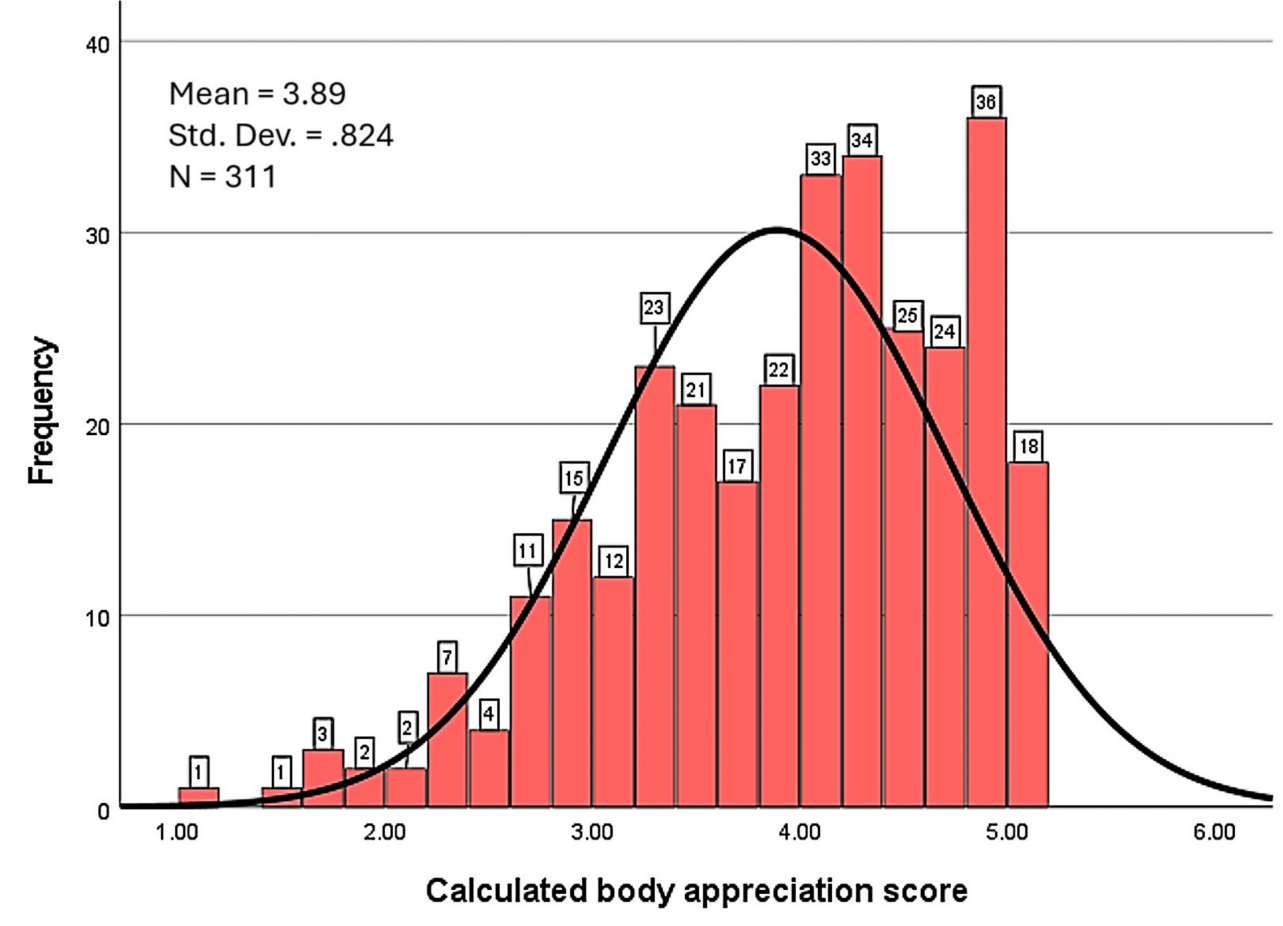
Histogram of body appreciation scores. Body appreciation scores were calculated based on the Body Appreciation Scale (BAS-2) [15].

Body appreciation scores [15], visualized in the histogram below (Figure 4), ranged from 1.10 to 5.00, with an average score of 3.89 (SD = 0.82). Most participants showed moderate to high levels of body appreciation, with 85.2% (n = 265) scoring between 3 and 5, and over half (50.5%) (n = 157) scoring in the highest range (4.10-5.00).

Correlation analysis displayed a statistically significant inverse relationship between body dissatisfaction and body appreciation. Due to non-normal distributions, Spearman's rank correlation was used, revealing a moderate negative correlation (r = −0.431, p < 0.001), indicating that higher body appreciation was associated with lower body dissatisfaction.

Eating disorder risk

**Figure 5:**
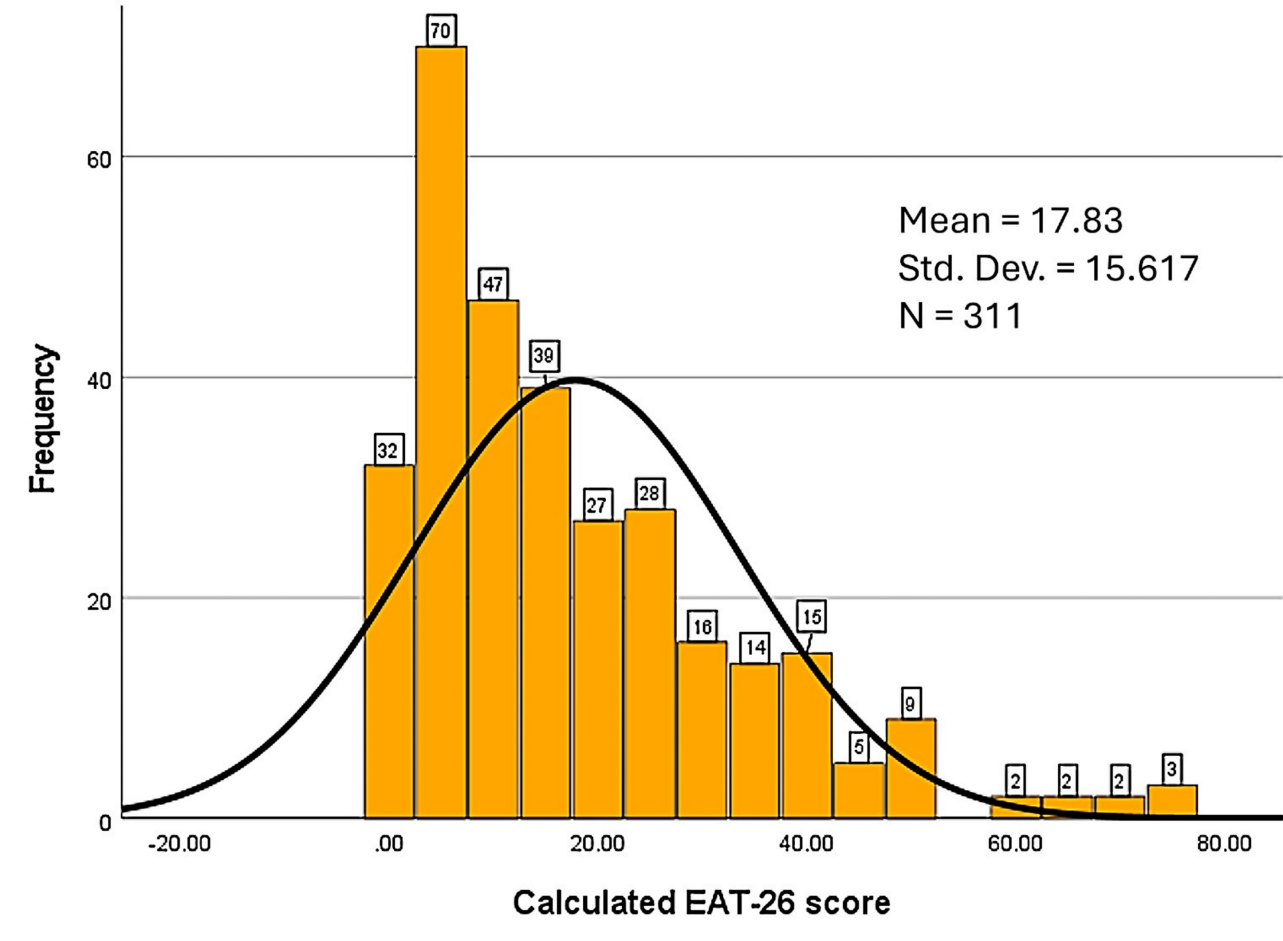
Histogram of EAT-26 scores. EAT-26: Eating Attitudes Test (26 referring to the number of items in the instrument).

Eating disorder risk was assessed using the EAT-26 [16] scale. Total scores ranged from 0 to 75, with an average score of 17.83. Overall, 65.6% (n = 204) of participants scored below the risk cutoff of 20, indicating low to no risk of an eating disorder. However, 34.4% (n = 107) of participants were considered as being at risk, with a minority (4.1%) (n = 13) with very high scores (>50), reflecting considerable risk. This was visualized in the histogram in Figure 5.

Eating disorder risk and associated factors

The associations between eating disorder risk and the examined demographic and psychosocial factors are presented in Table 2. Sex was not significantly associated with eating disorder risk (p-value = 0.603), with comparable proportions of males and females classified as at risk. In contrast, several psychosocial factors showed strong associations with eating disorder risk.

Participants who reported that social media influenced their body perception were substantially more likely to be at risk compared to those who did not report any influence. Similarly, perceived pressure to modify one's body to meet media-driven ideals showed one of the strongest associations, with approximately half of the participants classified at risk, compared with those who did not report this pressure.

Interpersonal influences were also significant. Participants who reported receiving appearance-related remarks from family members or peers had more than double the prevalence of eating disorder risk compared with those who did not. Pandemic-related changes were likewise associated with eating disorder risk; participants who reported increased awareness of their eating behavior during COVID-19 lockdowns showed nearly three times the prevalence of risk compared with those who reported no change. 

**Table 2:** Eating disorder (ED) risk and associated factors. Statistical significance was defined as p < 0.05. Chi-square test was used for all two-category comparisons.

Factor	Category	ED risk n (%)	Low to no risk n (%)	p-value (significant if <0.05)
Sex	Male	30 (32.3%)	63 (67.7%)	0.603
	Female	77 (35.3%)	141 (64.7%)	
Social media influence	Yes	95 (40.3%)	141 (59.7%)	<0.001
	No	12 (16.0%)	63 (84.0%)	
Pressure from cultural beauty standards	Yes	78 (45.1%)	95 (54.9%)	<0.001
	No	29 (21.0%)	109 (79.0%)	
Pressure from family/peers	Yes	87 (41.2%)	124 (58.8%)	<0.001
	No	20 (20.0%)	80 (80.0%)	
COVID-19 lockdown	Yes	92 (43.2%)	121 (56.8%)	<0.001
	No	15 (15.3%)	83 (84.7%)	

Body dissatisfaction and associated factors

The associations between body dissatisfaction and the examined demographic and psychosocial factors are presented in Table 3. There was no statistically significant difference in body dissatisfaction scores between participants classified as at risk for an eating disorder and those at low or no risk (p = 0.069), although mean ranks were higher among at-risk participants (168.26 vs. 149.57). Similarly, body dissatisfaction scores did not differ significantly by sex (p = 0.491).

In contrast, but similar to the eating disorder risk, several psychosocial factors were significantly associated with higher body dissatisfaction scores. Participants who reported social media influence on body perception, appearance-related remarks from family or peers, and perceived pressure to conform to cultural beauty standards all demonstrated significantly higher dissatisfaction scores. 

Differences in body dissatisfaction were also observed in relation to perceived changes during the COVID-19 lockdown. Kruskal-Wallis (ANOVA) analysis revealed a statistically significant difference across groups reporting more positive, more negative, or no change in body perception during the pandemic (p = 0.009).

Overall, body dissatisfaction and eating disorder risk were prevalent among a notable minority of students. While demographic factors, such as sex, showed minimal association, psychosocial influences, including social media exposure, cultural and interpersonal pressures, and COVID-19 pandemic-related perception changes, were consistently significantly associated with both increased body dissatisfaction and increased eating disorder risk.

**Table 3:** Body image dissatisfaction (BID) and associated factors. Statistical significance was defined as p < 0.05. The Mann–Whitney U test was used for two-group comparisons, and the Kruskal–Wallis test for comparisons with more than two groups (COVID-19 lockdown effect). ED: eating disorder.

Factor	Category	N	Mean rank	p-value (significant if <0.05)
ED risk	At risk	107	168.26	0.069
	Low to no risk	204	149.57	
Sex	Male	98	161.15	0.491
	Female	218	153.81	
Social media influence	Yes	236	166.81	<0.001
	No	75	121.97	
Pressure from cultural beauty standards	Yes	173	171.09	0.001
	No	138	137.08	
Pressure from family/peers	Yes	211	171.33	<0.001
	No	100	123.66	
COVID-19 lockdown	Yes	101 (more positive)-111 (more negative)	139.82 (more positive)-175.09 (more negative)	0.009
	No	99	151.11	

## Discussion

The purpose of the present study is to explore the relationship between body image dissatisfaction (BID) and disordered eating attitudes in University of Sharjah students in the United Arab Emirates and investigate the influence of external factors on the aforementioned variables. The majority of our participants' body dissatisfaction scores were low, which corresponded with the overall high body appreciation scores. These results suggest an overall positive subjective evaluation of self/body image in our sample population. Moreover, the scores of the Eating Attitudes Test (EAT-26) [16] demonstrated an overall low risk of developing an eating disorder, as 65.6% (n = 204) of our participants scored below the cut-off for determining the risk. Notably, however, no statistically significant association was found between BID and sex. The association between BID and the risk of eating disorders was also not statistically significant, which disproves our hypothesis. Nevertheless, secondary analyses revealed significant associations with external factors, discussed subsequently.

COVID-19 lockdown effects

As we were working on the study towards the end of the COVID-19 lockdown, we believed it was important to explore the possible effects of COVID-19 on the body image and eating attitudes of our population. A dual effect was noted as approximately one-third of participants reported becoming more positively conscious of their bodies during that time, while another third experienced the contrary. Additionally, 68.5% (n = 213) were more conscious of eating behaviors during the pandemic lockdown. The heterogeneity of these results is likely a reflection of the effects of various factors on the individuals, as was reported in a recent systematic review [12]. During the lockdown, there was increased isolation for many, and the negative impact of these restrictions has been studied [17]. The increased time resulting from the isolation could lead to more time spent on social media [18], and for those who reported that social media influences their perception of their body (75.9% in our study, n = 236), this may lead to increased cognizance of body image. That heightened awareness could impact the body image positively, via body-positive content [19,20] or negatively, as proposed by the social comparison theory [1,21,22] and other studies [18].

Social interactions and body perception

Despite the isolating nature of the lockdown, many spent increased time in close proximity to others, depending on their living situations. 67.8% (n = 211) of respondents reported that remarks from others around them influenced their perceptions of their bodies. Increased time with those who do leave these remarks may lead to changes in their self/body perception as well as eating attitudes/behaviors, as studies show that our social circle is an influential factor in body image [23-28]. Our analysis also found a significant association between these factors (social media, cultural beauty standards, and remarks from family/peers) with both body image dissatisfaction and risk of eating disorders.

Literature comparisons

These findings differ from much of the existing literature, which has reported higher levels of body dissatisfaction and stronger associations with disordered eating. Body dissatisfaction and disordered eating in adolescents and young adults have been rising significantly [3-6]. The absence of a statistically significant association between body dissatisfaction and disordered eating attitudes in this study may reflect the complex and multifactorial nature of these constructs. Body dissatisfaction does not necessarily translate directly into disordered eating behaviors, particularly in populations with higher health awareness and adaptive coping strategies, such as medical students. Additionally, the significant independent associations observed between external factors, such as social media influence, cultural beauty standards, and remarks from family or peers, and both body dissatisfaction and eating disorder risk suggest that these variables may act as mediators or moderators rather than body dissatisfaction serving as a direct predictor of disordered eating. Sampling characteristics and limited sample size may have further reduced the ability to detect subtle associations, underscoring the importance of considering contextual, cultural, and psychosocial factors when interpreting these findings.

United Arab Emirates (UAE)-specific context

Specifically in the UAE, previous studies explored these variables in one of the sexes [7,9,10], but not both, as was done in our research. In the first two of these studies, however, along with other international ones [29], a significant association was found between body dissatisfaction and disordered eating attitudes, in contrast to the null findings of the present study. Notably, however, a study done amongst UAE University female students [10] found that body dissatisfaction did not have a direct effect on disordered eating. This may suggest that the link between these variables is more complex. This finding could be affected by our limited sample, but it could indicate that the interplay between these variables may be multifactorial, as supported by their independent significant associations with the aforementioned external factors.

Implications for practice

Despite the absence of a statistically significant association between BID and ED risk, the findings have several important practical implications. The psychosocial impact of BID extends beyond eating habits, affecting self-esteem, interpersonal relationships, and mental health more broadly. Although these dimensions were not directly measured in our study, existing literature supports the role of BID in shaping emotional well-being, highlighting the need for a multidisciplinary approach that includes psychological support and health education in both academic and community settings. From an educational standpoint, interventions such as media literacy programs, peer-led workshops, and supportive campus mental health services could help mitigate the harmful effects of unrealistic beauty ideals. Integrating community-based awareness campaigns that celebrate body diversity and promote self-acceptance, particularly through accessible and engaging platforms, may further enhance resilience against BID and disordered eating behaviors, as well as prevent their development into clinical disorders. Medical professionals also play a pivotal role in this early detection and prevention. Routine health checkups, especially in primary care and student health centers, could help identify early signs of eating disorders and initiate timely psychological counseling.

Directions for future research

Future research should focus on longitudinal studies to track the evolution of body image and eating attitudes over time, particularly in response to ongoing exposure to media and societal pressures. Studies with larger, more diverse, and more representative samples will help improve generalizability and clarify gender-related differences. In-depth qualitative studies exploring external influences, such as peer dynamics, family attitudes, and media exposure, may also enrich our understanding of these complex relationships. Finally, comparative research across different cultural and socioeconomic groups could offer insight into how contextual factors mediate the relationship between BID and ED risk.

Strengths and limitations

Strengths

This study provides valuable insight into body image dissatisfaction and eating attitudes among university students in the United Arab Emirates, a population that remains relatively underrepresented in regional and international literature. One of the primary strengths of this research is the use of validated and widely recognized assessment instruments, including the Figure Rating Scale for Body Image Dissatisfaction (BID) [14], Body Appreciation Scale-2 (BAS-2) [15], and Eating Attitudes Test-26 (EAT-26) [16], which enhances the reliability of the findings and allows for meaningful comparison with previous studies. Furthermore, the inclusion of a broad range of psychosocial and environmental variables, including social media exposure, cultural beauty standards, peer and family influence, and COVID-19-related behavioral changes, provides a multidimensional perspective on factors associated with eating attitudes and body image perceptions.

Limitations

Several limitations should be acknowledged. Data collection relied on self-reported measures, which are inherently subject to reporting bias, including social desirability and recall bias, particularly when addressing sensitive topics such as eating behaviors and body image. In addition, the study was conducted at a single academic institution, which may limit the generalizability of the findings to other university populations or to young adults outside the academic setting. The use of a non-probability volunteer sampling method may have introduced selection bias, as students with greater awareness or interest in health-related topics may have been more inclined to participate. Moreover, the sample included a relatively high proportion of students enrolled in medical and health-related disciplines, who may differ from the general student population in terms of health knowledge and coping strategies. Lastly, the cross-sectional design of the study limits the ability to establish temporal or causal relationships between body image dissatisfaction, eating attitudes, and associated psychosocial factors. As such, the observed associations should be interpreted as correlational, and conclusions regarding directionality or causation cannot be drawn. However, the use of validated and widely utilized instruments (EAT-26 and BAS-2) and the anonymous nature of survey administration may have helped mitigate some of these biases. Future research could utilize objective assessments and/or clinical interviews to study the associations between eating attitudes and body image in a more comprehensive manner.

## Conclusions

The findings of this study highlight the prominent roles of psychosocial and environmental influences, particularly social media exposure, cultural beauty ideals, and peer and family remarks, in relation to eating attitudes among university students. Despite generally low levels of body image dissatisfaction within the study population, a considerable proportion of participants were identified as being at risk of disordered eating, underscoring the importance of addressing this issue within young adult populations.

Although no statistically significant association was observed between body image dissatisfaction and eating disorder risk, the results suggest that eating attitudes may be shaped by a complex interplay of social and contextual factors rather than body dissatisfaction alone. These findings emphasize the need for preventive and educational initiatives that promote healthy body image, media literacy, and awareness of the potential psychological and behavioral consequences of unrealistic beauty standards. Future studies employing longitudinal designs and more diverse, multi-institutional samples are recommended to further clarify these relationships and to better inform targeted intervention strategies.

## Appendices

Questionnaire 

Below is a text version of the self-administered online questionnaire used for participant enrollment and data collection in the present study. The questionnaire was developed and administered by the study authors and distributed electronically via Google Forms.

The demographic items (Table 4) were developed specifically for this study by the authors. Validated instruments incorporated into the questionnaire included the Body Image Dissatisfaction (BID) figure rating scale [14] (Figure 1, Table 5), the Body Appreciation Scale-2 (BAS-2) [15], and the Eating Attitudes Test-26 (EAT-26) [16], which were adopted from previously published sources and are cited in the references accordingly [14-16]. No modifications were made to the original scoring or interpretation of these instruments.

**Table 4:** Demographics. Created by Authors.

Variable	Question	Response options
Sex	What is your sex?	Male/female
Age	What is your age?	Numeric (years)
Nationality	What is your nationality?	Free-text response
College	Select which college you are currently in	Sharia and Islamic Studies, Arts/Humanities & Social Sciences, Business Administration, Engineering, Health Sciences, Law, Fine Arts & Design, Communication, Medicine, Dental Medicine, Pharmacy, Sciences, Computing & Informatics

**Table 5:** Body image dissatisfaction (BID) figure rating scale. Body images/figures referenced are available in Figure 1.

Instrument	Item	Response options
Figure rating scale [14]	Please select which figure is most similar to your actual body.	Eight-point figure scale (Body 1 = smallest body size to Body 8 = largest body size)
Figure rating scale [14]	Please select which figure is most similar to your ideal body.	Eight-point figure scale (Body 1 = smallest body size to Body 8 = largest body size)

In addition, psychosocial factor questions (Table 6), including items related to social media, culture, family/peers, and COVID-19 lockdown-related perception changes, were also developed specifically for this study by the authors.

**Table 6:** Psychosocial factors related to Body Image Dissatisfaction (BID) and Eating Disorder (ED) risk. Created by Authors.

Variable	Question	Response options
Social media influence	Do you feel like social media influences your perception of your body?	Yes/No
	Do you feel the need to take measures (i.e. cosmetic procedures, extreme dieting/exercise) to achieve a certain body type you often see in the media?	Yes/No
Culture	Have you ever felt pressured to fit into your culture’s beauty standard?	Yes/No
Social circle	Do the people around you (family/peers) leave remarks that influence your perception of your body?	Yes/No
COVID-19 lockdown	Has the pandemic (lockdown) made you more conscious of your body?	Yes, more positive Yes, more negative No
	Has the pandemic (lockdown) made you more conscious of your eating behaviors?	Yes/No

Body Appreciation (BAS-2)

The Body Appreciation Scale-2 (BAS-2) [15] is a validated self-report instrument used to assess positive body image. The scale consists of 10 items rated on a five-point Likert scale, with higher scores indicating greater body appreciation.

In accordance with journal guidance, the full item content of the BAS-2 [15] is not reproduced in this Appendix. The scale was administered in its original, unmodified form, and an appropriate citation to the source has been provided in the Methods section and reference list.

Eating Attitudes (EAT-26)

The Eating Attitudes Test-26 (EAT-26) [16] is a validated self-report instrument for assessing the risk of eating disorders. The questionnaire consists of 26 items rated on a six-point Likert scale, with a total score ≥20 indicating elevated risk for disordered eating behaviors.

In accordance with copyright guidelines provided by the instrument’s copyright holder [16], the full item content of the EAT-26 [16] is not reproduced in this Appendix. The scale was administered in its original, unmodified form, and an appropriate citation to the source has been provided in the Methods section and reference list.
